# Single-cell analysis of progenitor cell dynamics and lineage specification in the human fetal kidney

**DOI:** 10.1242/dev.164038

**Published:** 2018-08-30

**Authors:** Rajasree Menon, Edgar A. Otto, Austin Kokoruda, Jian Zhou, Zidong Zhang, Euisik Yoon, Yu-Chih Chen, Olga Troyanskaya, Jason R. Spence, Matthias Kretzler, Cristina Cebrián

**Affiliations:** 1 Department of Computational Medicine and Bioinformatics, University of Michigan, Ann Arbor, MI 48109, USA; 2 Department of Internal Medicine, Division of Nephrology, University of Michigan, Ann Arbor, MI 48109, USA; 3 Department of Internal Medicine, Division of Gastroenterology, University of Michigan, Ann Arbor, MI 48109, USA; 4 Lewis-Sigler Institute for Integrative Genomics, Princeton University, Princeton, NJ 08544, USA; 5 Graduate Program in Quantitative and Computational Biology, Princeton University, Princeton, NJ 08544, USA; 6 Department of Electrical Engineering and Computer Science, Department of Biomedical Engineering, University of Michigan, Ann Arbor, MI 48109, USA; 7 Flatiron Institute, Simons Foundation, New York, NY 10010, USA; 8 Department of Computer Science, Princeton University, Princeton, NJ; 9 Department of Cell and Developmental Biology, Division of Gastroenterology, University of Michigan, Ann Arbor, MI 48109, USA

**Keywords:** Branching, Fetal, Human, Kidney, Single cell

## Abstract

The mammalian kidney develops through reciprocal interactions between the ureteric bud and the metanephric mesenchyme to give rise to the entire collecting system and the nephrons. Most of our knowledge of the developmental regulators driving this process arises from the study of gene expression and functional genetics in mice and other animal models. In order to shed light on human kidney development, we have used single-cell transcriptomics to characterize gene expression in different cell populations, and to study individual cell dynamics and lineage trajectories during development. Single-cell transcriptome analyses of 6414 cells from five individual specimens identified 11 initial clusters of specific renal cell types as defined by their gene expression profile. Further subclustering identifies progenitors, and mature and intermediate stages of differentiation for several renal lineages. Other lineages identified include mesangium, stroma, endothelial and immune cells. Novel markers for these cell types were revealed in the analysis, as were components of key signaling pathways driving renal development in animal models. Altogether, we provide a comprehensive and dynamic gene expression profile of the developing human kidney at the single-cell level.

## Abstract (precis)

**Summary:** New markers for specific cell types in the developing human kidney are identified and computational approaches infer developmental trajectories and interrogate the complex network of signaling pathways and cellular transitions.

## INTRODUCTION

The development of the embryonic kidney is a paradigm for the complex inductive and regulatory mechanisms driving organogenesis (Grobstein, 1953). In mammals, the epithelial ureteric bud (UB) tips undergo repetitive branching morphogenesis until birth and generate the entire collecting system, including the ureter, calyces and collecting ducts, but not the bladder (Chi et al., 2009; Riccio et al., 2016; Shakya et al., 2005). Around the tips of the UB, nephron progenitors condense into cap mesenchyme, epithelialize and undergo complex morphogenesis to differentiate into the vast majority of cells in the nephron, including parietal epithelial cells, the podocytes, proximal and distal tubules, loops of Henle and the connecting tubule (Cebrian et al., 2014; Kobayashi et al., 2008; Mugford et al., 2008). Interspersed between the nephron progenitors, the stroma promotes survival and differentiation of the progenitors as well as branching of the UB and contribute to the mesangial and endothelial lineage (Das et al., 2013; Mugford et al., 2008).

Studies in mice have identified several signaling pathways involved in the reciprocal epithelial-mesenchymal interactions driving kidney development. For example, soluble ligands are expressed by the mesenchymal nephron progenitors and signal to receptors in the epithelial UB tip to activate downstream transcription factors. Reciprocally, the UB tip cells also release signals to the mesenchyme to maintain its self-renewal. These signaling cascades are crucial for UB outgrowth and branching, and involve a host of morphogens and signaling networks, including Gdnf-Ret, Wnt, Fgf and Notch signaling, to name a few (Cacalano et al., 1998; Lu et al., 2009; Pachnis et al., 1993; Pichel et al., 1996; Schuchardt et al., 1994). Collectively, these signaling events regulate the balance between self-renewal and differentiation of nephron progenitors (Carroll et al., 2005; Park et al., 2007), ensure proper positioning of the metanephric mesenchyme (MM) and UB outgrowth (Huang et al., 2014; Pietilä et al., 2016; Yun and Perantoni, 2017), and promote further differentiation into specific cell types (Cheng et al., 2007; Surendran et al., 2010).

Although the murine and the human kidney seem to share a common developmental pattern, they also present anatomical, physiological and pathophysiological differences, as well as significant differences in gene expression (O'Brien et al., 2016), suggesting that significant new information can be gained by directly studying human kidney development. Here, we have used single-cell RNA-sequencing to interrogate 6414 cells from five developing human kidneys and have identified distinct cell populations based on their gene expression profile. These populations include progenitors, immature and mature renal cell types and proliferating cells. We further identify new markers for specific cell types in the developing human kidney, and we use computational approaches to infer developmental trajectories and interrogate the complex network of signaling pathways and cellular transitions during development.

## RESULTS

### Single-cell sequencing identifies progenitors, and intermediate and mature cell types in the developing human kidney

**Fig. 1.**
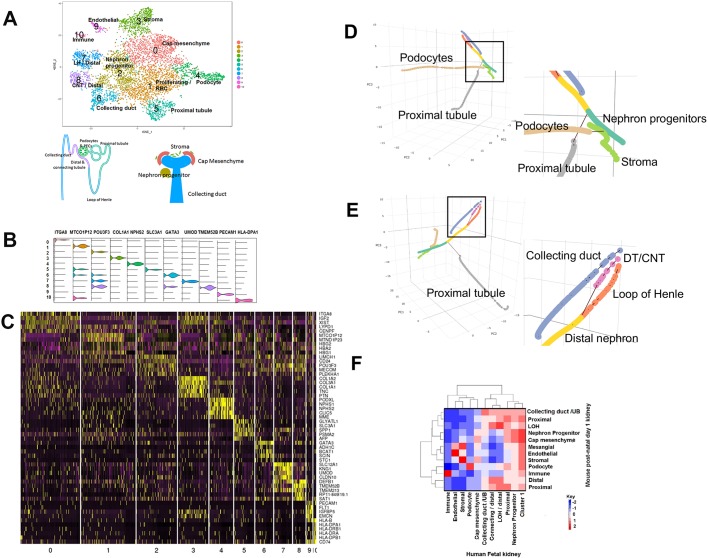
**Human fetal kidney single cell types.** (A) tSNE plot showing the 11 cell clusters from the combined analysis of the five fetal kidney datasets. A total of 6414 cells with at least 500 genes per cell were identified. The diagram depicts developing and mature renal structures identified in the tSNE plot. The number of cells per cluster is as follows: 0 (1384), 1 (1203), 2 (895), 3 (662), 4 (568), 5 (430), 6 (406), 7 (395), 8 (270), 9 (135), 10 (66). (B) Violin plots showing the expression pattern of a top differentially expressed (cluster-specific) gene from each of the 11 clusters. (C) Heatmap with the expression pattern of the top five cluster-specific genes in 11 clusters. (D,E) Two different perspectives of the lineage trajectory analysis of single-cell RNAseq data from human embryonic kidney. Boxed area highlights the initial branch out of podocytes and proximal tubules from the nephron progenitor lineage. Boxed area in E highlights the branch out of distal tubules (DT) and connecting tubules (CNT) from the loop of Henle. (F) Heatmap showing the correlation between the average expression of all genes expressed in human fetal kidney cell clusters to mouse postnatal day 1 kidney clusters. The color intensity in the heatmap is based on the z-scores from the row-wise scaling of the correlation matrix.

Analysis of single-cell sequencing data of 6414 cells from five human embryonic kidneys revealed 11 clusters (Fig. 1A-C and Table S1). The cellular identity of these clusters was assigned based on a list of representative genes most significantly expressed in these single cells (Table 1). A violin plot showing the expression of a representative gene and a heat map with the expression of the five most significantly differentially expressed genes for each cluster are shown in Fig. 1B and C, respectively. This analysis identifies most of the cell types expected in a developing kidney, from undifferentiated cap mesenchyme to differentiated epithelia (podocytes, proximal tubules, loops of Henle, collecting ducts and distal/connecting tubules), stroma, endothelial and immune cells as well as erythrocytes. In addition to these differentiated cell types, cluster 2 represents a population of cells with gene expression that is characteristic of the developing nephrons. The QC parameters indicated similarities between the five datasets (Fig. S1A,B). Fig. S1C,D shows clearly that the 11 clusters contained cells from all five datasets. It was interesting to observe higher level of mitochondrial read content in cluster 7 (loop of Henle) compared with all other clusters (Fig. S1E).

**Table 1.**
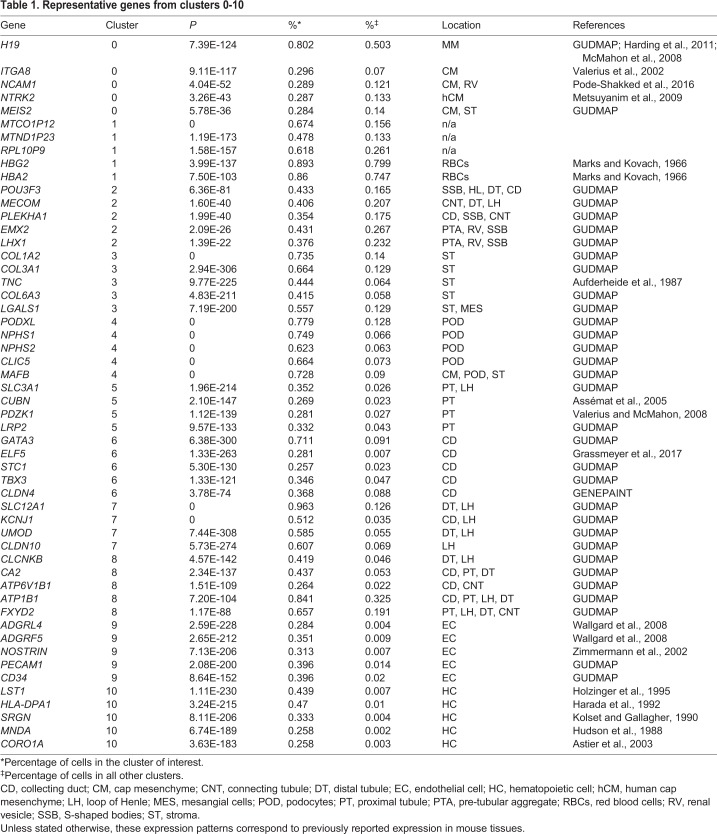
**Representative genes from clusters 0-10**

tSNE (t-distributed stochastic neighbor embedding) dimensionality reduction identifies progenitor and differentiated cell types in 2D but fails to identify lineage relationships among different clusters. Therefore, to inquire whether single-cell data can be used to identify lineage relationships, we have performed pseudotemporal trajectory analysis, which uses an unbiased computational approach to infer developmental trajectories and distinguish between progenitor cell types in 3D (Fig. 1D,E and Movie 1). This approach identifies three independent developmental trajectories: the stroma, the collecting duct and the nephron lineage. For each trajectory, immature cell types are positioned towards the center of the plot whereas more mature cell types are positioned towards the periphery. Differentiated cell types branch out from the nephron trajectory (shown as black lines in Fig. 1D,E); interestingly, the podocytes are the first ones to emerge from the nephron progenitors (see insert box in Fig. 1D) followed by the proximal and distal segments. The distal nephron further differentiates into loop of Henle and distal/connecting tubules (insert box in Fig. 1E). Altogether, our approach identifies cell populations and developmental trajectories that are consistent with the major renal mature cell types, their progenitors and intermediate stages of differentiation.

To further validate our analysis, we have clustered single-cell sequencing data from postnatal day 1 mice (Adam et al., 2017) and compared those clusters (Fig. S1F) with the ones generated from human single-cell sequencing. As shown in Fig. 1F, mouse and human clusters show significant correlation. Therefore, despite differential expression of specific genes, the overall gene expression and cellular identity between the mouse and human developing kidney is highly conserved. Interestingly, geneontology enrichment analysis of the significantly expressed genes in each cluster compared with all other clusters showed relevant biological processes for the identified cell types (Fig. S1G). For example, cell-cell adhesion and extracellular matrix organization were among the top terms for podocytes.

### Sub-clustering of undifferentiated and nephron progenitor cells reveals several maturation stages

**Fig. 2.**
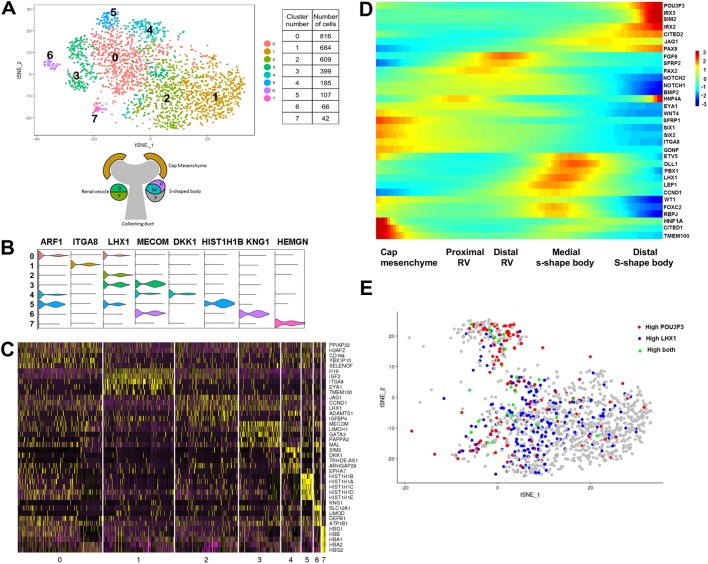
**Sub-clustering of nephron progenitor cells.** (A) tSNE plot showing the eight clusters from the sub-clustering of 0, 1 and 2 clusters from the initial clustering analyses. The diagram depicts developing nephron structures identified in the tSNE plot. The table provides the number of cells found in each cluster. (B) Violin plots showing the expression pattern of a top differentially expressed (cluster-specific) gene from eight sub-clusters of the original clusters 0, 1 and 2. (C) Heatmap with the expression pattern of the top five cluster-specific genes in the eight sub-clusters of the original clusters 0, 1 and 2. (D) Heatmap showing gene expression dynamics of signaling molecules and cell-specific markers in 1, 2 and 4 sub-clusters (cap-mesenchyme, proximal and distal nephron) of the original clusters 0, 1 and 2. The heatmap was generated using Monocle. Genes (row) are clustered and cells (column) are ordered according to the pseudotime development. (E) Feature plot of *LHX1* and *POU3F3* in 1, 2 and 4 sub-clusters (cap-mesenchyme, proximal and distal nephron) of the original clusters 0, 1 and 2. Cells with high expression of *POU3F3* are in red and the cells with high expression of *LHX1* are in blue. Cells with high expression of both *POU3F3* and *LHX1* are in green. The FeaturePlot function in Seurat R package that shows co-expression of two genes was used to generate this plot. According to this function, for each gene, the cells are divided into two groups (intervals) of equal size based on the range of gene expression using ‘cut’ R function. The group with higher expression is designated as ‘high’.

The initial clustering analysis shown in Fig. 1A identified discrete differentiated cell populations consistent with the existence of specific mature renal cell types in the human embryonic kidney (clusters 3-9). In addition, a large population of cells with the expression pattern of renal progenitors was also identified (clusters 0-2). On the other hand, the intermediate stages that are anatomically described as renal vesicles, pre-tubular aggregates, and comma- and s-shaped bodies were not distinctly identified in this initial clustering. To gain further insight into the gene expression signature of these intermediate stages, we have performed sub-clustering of clusters 0-2, as shown in Fig. 2. A total of eight clusters were identified and shown in a tSNE plot (Fig. 2A and Table S2). A violin plot showing the expression of a representative gene and a heat map with the expression of the five most significantly differentially expressed genes for each cluster are shown in Fig. 2B and C, respectively. By analyzing cell specificity of the most significantly differentially expressed genes in these clusters (Table 2), we can begin to unravel the cellular identity of these clusters. Fig. S2A,B shows the distribution of cells from the five datasets in these eight sub-clusters.

**Table 2.**
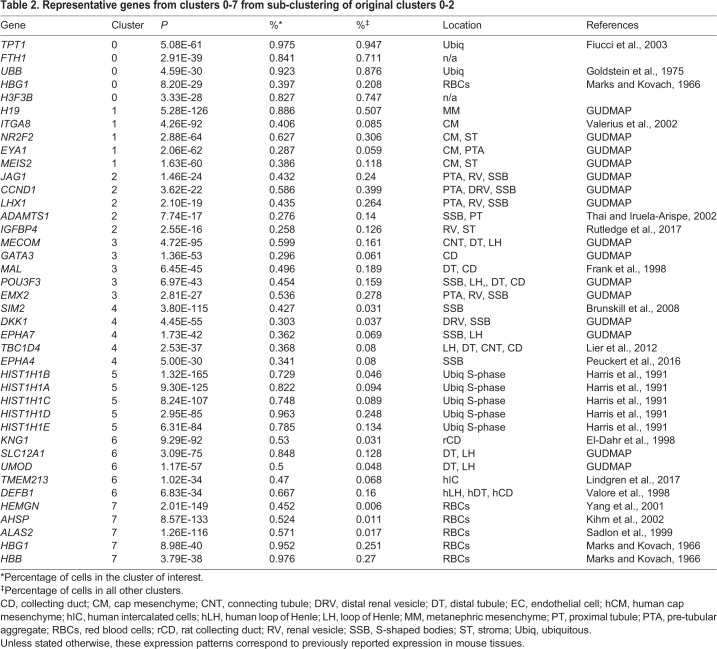
**Representative genes from clusters 0-7 from sub-clustering of original clusters 0-2**

Sub-cluster 0 contains cells that express ubiquitous markers, erythrocyte markers, nephron progenitor markers (see *LHX1* in Fig. 2B violin plot) as well as various proliferation markers, including *CCND1*, *UBE2C*, *TOP2A*, *MKI67*, *CDK4* and *CDK6* (Fig. S2C). In addition, cells in sub-cluster 5 present elevated expression of H1 linker histones; the mRNA level of these histones is greatly increased as cells progress from G1 to S phase, indicating that cells in sub-cluster 5 are undergoing mitosis (Harris et al., 1991). In addition, cells in sub-cluster 7 express genes associated with developing and mature erythrocytes, consistent with them being embryonic red blood cells. Sub-cluster 1 is defined by the expression of *ITGA8* and *EYA1*, reported markers of the nephrogenic mesenchyme in mice, the undifferentiated murine nephron progenitors (Kalatzis et al., 1998; Valerius et al., 2002). The expression pattern in cells in sub-cluster 2 is consistent with cells that have already initiated the differentiation process, with the expression of *JAG1* and *LHX1*; both these genes are markers of the pre-tubular aggregate in mice and humans (Crosnier et al., 2000; Fujii et al., 1994; McCright et al., 2001). Sub-cluster 3 contains cells with a more distal profile, with the expression of genes such as *MECOM*, *POU3F3* and *GATA3*; similarly, sub-cluster 6 contains distal nephron cells with an expression profile consistent with a loop of Henle identity. Finally, cells in sub-cluster 4 express *SIM2* and *EPHA4*, genes that, in mouse, are expressed by the s-shaped bodies (Brunskill et al., 2008; Peuckert et al., 2016).

To gain further insight into the dynamic gene expression of early nephron progenitors as they differentiate, we have plotted the expression of relevant genes in cells from sub-clusters 1 (cap mesenchyme), 2 (pre-tubular aggregates) and 4 (s-shaped bodies). The resulting heat map is shown in Fig. 2D. This analysis reveals distinct populations of cells based on the expression of these marker genes. The expression of *CITED1*, *TMEM100*, *SFRP1*, *SIX1*, *SIX2*, *WT1*, *ITGA8*, *HNF1A* and *GDNF* is consistent with these cells being cap mesenchyme. Most of these genes are also expressed, but at lower levels, in a second population of cells characterized by the expression of *HNF4A*, *EYA1* and *PAX2*; this expression profile is consistent with these cells being part of the proximal renal vesicle. A third population is characterized by the expression of *FGF8* and *SFRP2*, which in mouse are expressed in the distal segments of the renal vesicles and comma-shaped bodies (Grieshammer et al., 2005; Heliot et al., 2013; Leimeister et al., 1998). Finally, two more populations are identified: one corresponding to the distal segment of the s-shaped body expressing *POU3F3* (Lindstrom et al., 2018b) and one corresponding to the medial segment of the s-shaped body expressing *FOXC2* and *LHX1* (Lindstrom et al., 2018b). We have plotted the expression of *POU3F3* and *LHX1* on 1, 2 and 4 sub-clusters (cap mesenchyme, proximal and distal nephron) of the original clusters 0, 1 and 2 (Fig. 2E). The cells with higher *POU3F3* expression cluster away from those of high *LHX1* expression, further confirming that they belong to different segments (distal and medial) of the developing nephron.

### Parietal epithelial cells and immature and mature podocytes present distinct gene expression profiles in the developing human kidney

**Fig. 3.**
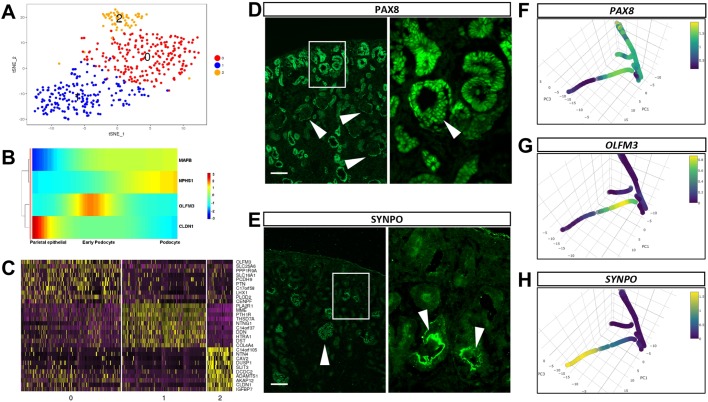
**Podocyte maturation and trajectory.** (A) tSNE plot showing the three clusters from the sub-clustering of cluster 4 (podocyte like) from the initial clustering analyses. (B) Heatmap showing the expression levels of *MAFB*, *NPHS1*, *OLFM3* and *CLDN1* in parietal epithelial, early podocyte and mature podocyte cells. (C) Heatmap with the expression pattern of the top 10 cluster-specific genes in the three sub-clusters of the original cluster 4. (D) Immunofluorescent assay detecting PAX8 localization in the human embryonic kidney. Arrowheads indicate mature glomeruli where PAX8 is expressed in the parietal epithelial cells. Arrowhead in the high-magnification inset indicates an immature glomerulus where PAX8 is located in both the parietal epithelial cells and the developing podocytes. Scale bar: 100 µm. Representative image from at least three independent stainings. (E) Immunofluorescent assay detecting SYNPO localization in the human embryonic kidney. Strongest expression is detected in mature glomeruli (arrowheads). Scale bar: 100 µm. Representative image from at least three independent stainings. (F-H) Expression pattern of *PAX8* (F), *OLMF3* (G) and *SYNPO* (H) shown along the trajectory path of podocyte development.

We have also performed sub-clustering analysis of the initial cluster 4, which is characterized by the specific expression of *PODXL*, *NPHS1*, *NPHS2* and *CLIC5*, and we first identified these cells as podocytes (Table 1). Fig. 3A-C shows the tSNE plot of this sub-clustering and heatmap of expression of representative genes; the full list of genes is provided in Table S3. The tSNE plot showing the distribution of cells from each embryonic sample demonstrates contribution from all samples to the three identified clusters (Fig. S2D,E). Cells in sub-cluster 2 are characterized by the specific expression of known parietal epithelial cells such as *CAV2*, *PTRF* (*CAVIN1*), *CLDN1* and *CLDN3* (Kiuchi-Saishin et al., 2002; Krawczyk et al., 2017; Ohse et al., 2008). Cells in sub-cluster 1 express *PODXL*, *NPHS1*, *NPHS2*, *SYNPO* and *VEGF*, all markers of mature human podocytes (Bariety et al., 2006). We have plotted the expression of *NPHS1* and *CLDN1* in this sub-clustering (Fig. S2F), showing that cells in sub-cluster 2 have high expression of *CLDN1* alone or in combination with *NPHS1*, an expression profile compatible with their identity as parietal epithelial cells. Sub-cluster 0 is populated by cells that express specifically *OLFM3*. When we plot the expression of this gene on the trajectory analysis (Fig. 3G), we observe that this gene is not only specific for the podocyte trajectory, but it is also restricted to the more immature section of it. The PAX8 protein has been detected in developing podocytes by immunohistochemistry, but is absent from the mature podocytes that are instead positive for mature marker SYNPO (Fig. 3D,E). *OLFM3* expression pattern overlaps with the immature podocyte marker *PAX8* (Fig. 3F) but is excluded from the mature podocytes that express *SYNPO* (Fig. 3H).

A number of PDZ domain proteins are expressed in the mature podocyte cluster: *HTRA1*, *MAGI2*, *SLC9A3R2*, *AHNAK*, *PDLIM2*, *PARD3B*, *PDLIM5*, *MPP5* and *TJP1*). Sub-cluster 1 (from sub-clustering of initial cluster 4) enrichment in PDZ domain proteins is consistent with a role for these proteins in establishing cell-cell contacts and the slit diaphragm that is characteristic of mature podocytes. Indeed, *MAGI2*, *PDLIM2*, *MPP5*, *PARD3B* and *TJP1* are associated with cytoskeletal and barrier function in podocytes (Ihara et al., 2014; Itoh et al., 2014; Koehler et al., 2016; Lu et al., 2017a; Sistani et al., 2011).

### Sub-clustering of the stromal cluster identifies mesangial cells, as well as cortical and medullary stroma

**Fig. 4.**
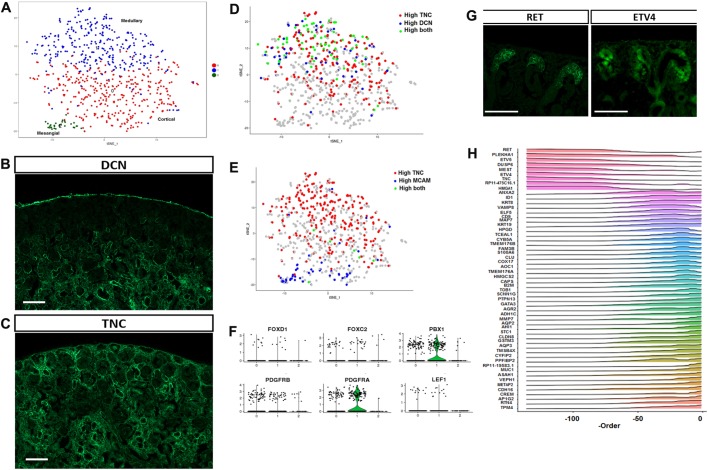
**Stromal and collecting duct cell types.** (A) tSNE plot showing the three sub-clusters of cluster 3 (stromal) from the initial clustering. (B) Immunofluorescence assay showing decorin expression exclusively in the medullary stroma of the human developing kidney. Scale bar: 100 µm. Representative image from at least three independent stainings. (C) Tenascin C protein is detected by immunofluorescence in both cortical and medullary areas of the human embryonic kidney. Scale bar: 100 µm. Representative images from at least three independent stainings. (D) Feature plot of TNC and DCN expression in the three sub-clusters of original cluster 3 (stromal). Cells with high expression of TNC are in red and the cells with high expression of DCN are in blue. Cells with high expression of both TNC and DCN are in green. The FeaturePlot function in Seurat R package that shows co-expression of two genes was used to generate this plot. According to this function, for each gene, the cells are divided into two groups (intervals) of equal size based on the range of gene expression. The group of cells with higher expression is labeled as ‘High’. (E) Feature plot of TNC and MCAM expression in the three sub-clusters of original cluster 3 (stromal). Cells with high expression of TNC are in red and the cells with high expression of MCAM are in blue. Cells with high expression of both TNC and MCAM are in green. (F) Violin plots depicting the expression levels of stromal genes, including *FOXD1*, *FOXC2*, *PBX1*, *PDGFRB*, *PDGFRA* and *LEF1*, in the three sub-clusters of cluster 3 (stromal) from the initial clustering. High expression of *FOXD1* and *FOXC2* was observed in multiple cells of sub-clusters 0 and 1. None of the cells expressed *LEF1* in sub-cluster 2. (G) Immunofluorescent detection of the receptor tyrosine kinase RET and its downstream target ETV4 in the developing human kidney. Scale bar: 100 µm. Representative images from at least three independent stainings. (H) Gene expression heatmap for the collecting duct differentiation.

In order to identify specific cell types within the stromal compartment, we have performed sub-clustering of cluster 3 of our initial analysis. The tSNE plot in Fig. 4A identifies three clusters, with sample contribution and heatmap of specific gene expression shown in Fig. S3A-C. A full list of genes is provided in Table S4. Cells in sub-cluster 2 of this sub-clustering present an expression profile compatible with their identity as mesangial cells. Both *ACTA2* and *ANGPT2* expression is specific for sub-cluster 2, and these genes are markers of the mesangial cells in humans and mice, respectively (Lu et al., 2017b; Scheinman et al., 1976). We also find expression of *TAGLN* (transgelin), a marker of the migrating mesangial cells that is expressed during glomerular development in the rat embryonic kidney and in human and adult rat kidneys upon injury (Daniel et al., 2012).

Cells in sub-cluster 1 express *SFRP1*, *TNC*, *DCN* and collagens 3A1, 1A2 and 1A1 (*COL3A1*, *COL1A2* and *COL1A1*). This expression pattern confirms that these cells are renal stromal cells. Interestingly, both *SFRP1* and *DCN* are not only highly expressed in sub-cluster 1, but also are very specific to sub-cluster 1. On the other hand, *TNC* and the collagens are strongly expressed in sub-cluster 1 but they are also expressed in the other two sub-clusters. Indeed, immunofluorescent detection of DCN and TNC in the developing human kidney (Fig. 4B,C) reveals a wide distribution in the renal stroma for TNC but a restriction to the medullary area for DCN. When we plot the expression of *TNC* and *DCN* in the tSNE plot (Fig. 4D), those cells expressing high *DCN* (blue dots) or high *DCN* with high *TNC* (green dots) are predominantly located in sub-cluster 1, indicating that this cluster represents the medullary stroma population. When we plot the expression of *TNC* and *MCAM*, markers of stromal cells that, in mice, differentiate into PECAM-expressing endothelial cells (Halt et al., 2016), we observe that their expression is mutually exclusive (Fig. 4E). These cells also express markers of mesangial cells and their expression pattern is compatible with the defining markers of a reported population of resident mesenchymal stem cells in the human glomeruli (Bruno et al., 2009).

In addition, cells in sub-cluster 0 have few specific genes, including *POSTN* and *CXCL12*. *Postn* (periostin) is expressed in the cortical stroma of the newborn rat kidney (Ito et al., 2002), in the mouse it is expressed in the renal stroma and the ureteric mesenchyme (Sorocos et al., 2011), and is a key player in the progression of renal disease, mediating inflammation and fibrosis (Prakoura and Chatziantoniou, 2017). In a similar fashion, *Cxcl12* is expressed in the cortical stroma of the developing mouse kidney, surrounding the cap mesenchyme and pre-tubular aggregates (Takabatake et al., 2009). This gene expression pattern indicates that cells in cluster 0 correspond to the cortical stroma. In mice, *Foxd1* is expressed in the stromal progenitors located in the cortex of the developing kidney and it is crucial for proper kidney development (Hatini et al., 1996). *FOXD1* expression is detected in a few of the human embryonic kidney cells in both clusters 0 and 1 (Fig. 4F). *PBX1* and *PDGFRA* are expressed at high levels in cluster 1; *Pbx1* is expressed in the developing and mature stroma of the embryonic mouse kidney, in perivascular cells along endothelia (Hurtado et al., 2015), whereas *PDGFRA* is expressed in the developing mouse kidney stroma and in kidney organoids from human iPS cells (Harding et al., 2011; McMahon et al., 2008; Takasato et al., 2015). The reduced number of cells expressing *FOXD1* in our analysis is likely due to a methodological cause, rather than reflecting a biological difference, as *FOXD1* has been shown to be expressed in the stromal progenitors in the developing human kidney (Lindstrom et al., 2018a).

### The human collecting system maintains a tip/trunk identity

We have performed single-cell sequencing on human embryonic samples using fragments spanning from the nephrogenic zone to the inner medulla. Hence, we expect to identify cells from the collecting duct lineage that span from the progenitor UB tip cells to the mature cells of the collecting duct. Sub-clustering of the original collecting duct cluster 6 identifies three distinct clusters (Fig. S3D-G and Table S5). Cells in sub-cluster 0 express *LHX1*, which in mice is expressed both in the developing nephrons as well as in the UB (Kobayashi et al., 2005). Cells in sub-cluster 1 express *CALB1*, which is expressed in the human collecting duct but mainly excluded from the tip (Fig. S3I). Finally, sub-cluster 2 includes a small number of cells that express *KRT7*, *UPK1A* and *KRT19*, all markers of mature collecting duct cells. When we plot the expression of known markers of the UB and the collecting duct onto the tSNE plot from this sub-clustering, we confirm that nearly all cells express *GATA3*, whereas *SPINK1* marks the mature collecting duct cells and *CALB1* and *RET* expression show little overlap (Fig. S3H), consistent with their expression in the trunk and the tip of the UB, respectively. To further investigate the expression pattern of RET and its downstream targets in human embryonic samples, we have performed immunofluorescence on human embryonic kidneys with specific antibodies against RET and ETV4 (Fig. 4G). RET is detected in the membrane of the cells at the tips of the branching UB, with some expression extending towards the trunk, and the ETV4 transcription factor is localized in the nucleus of the tip cells as well as in the early renal vesicles, an expression pattern that correlates with that of mouse embryonic kidneys (Lu et al., 2009). In addition, a gene expression heatmap for the collecting duct lineage generated using our trajectory analysis (Fig. 4H) identifies cells with expression of UB tip markers (*RET*, *ETV4*, *ETV5*, *DUSP6*) as well as cells expressing collecting duct markers (*GATA3*, *KRT8*, *KRT19*) and mature cells (*AQP2*, *MUC1*).

### Ligand-receptor analysis identifies known and novel signaling networks in the developing human kidney

In order to identify signaling networks between the different compartments of the developing human kidney, we have selected ligands or receptors that were significantly differentially expressed in individual clusters from our initial analysis and search for their ligand/receptor partners in other clusters. This analysis has revealed a list of signaling pathways shown in Fig. S4A.

*TNFSF10* [tumor necrosis factor (ligand) superfamily, member 10] is expressed in cluster 5, that corresponds to the proximal tubule cells. The TNFSF10 protein is localized to proximal and distal tubule cells in the adult human kidney (Song et al., 2004; Spierings et al., 2004). Like other tumor necrosis factors, TNFSF10 is involved in induced apoptosis as well as in inflammation, and in humans its increased expression is linked to the pathogenesis of diabetic nephropathy (Lorz et al., 2008).

Another group of signaling molecules identified in the analysis belong to the TGFβ superfamily and include *BMP7* as well as *TGFB2* and *TGFB3*. We observe significant differential expression of *BMP7* in cluster 4, which we have assigned a podocyte identity. In the mouse, *Bmp7* is expressed in the UB, the nephron mesenchyme, the developing nephron and the podocytes; removing *BMP7* from the podocytes during nephrogenesis results in hypoplastic kidneys and reduced proximal tubules (Kazama et al., 2008), suggesting a role for podocyte-specific BMP7 in promoting nephron differentiation and growth. TGFBR3 is an accessory receptor for the TGFB superfamily that is also significantly differentially expressed in the podocyte cluster. Previous studies have shown the expression of TGFBR3 in the developing mouse kidney and its role in establishing nephron endowment (Walker et al., 2011). Interestingly, TGFBR3 protein is strongly expressed in human adult podocytes (Uhlen et al., 2015) (www.proteinatlas.org/ENSG00000069702-TGFBR3/tissue/kidney#img). Very little is known, however, about its expression or role in podocyte development or physiology. On the other hand, TGFBR2 is expressed in the endothelial cluster (cluster 9). TGFBR2 is expressed in endothelial cells during the development of several organs, including the heart, the brain and the skin (Jiao et al., 2006; Nguyen et al., 2011; Robson et al., 2010; Yamazaki et al., 2017). In the mouse kidney, deletion of Tgfbr2 in the renal pericytes compromises myofibroblast recruitment (LeBleu et al., 2013).

NOTCH signaling is also identified by our analysis (Fig. S4B). *JAG1* is significantly differentially expressed in the proximal tubule cluster (cluster 5), *NOTCH4* is specifically expressed in the endothelial cells (cluster 9) and NOTCH2 is more widely expressed in the cap mesenchyme, the stroma and the podocytes (clusters 0, 3 and 4, respectively). This expression pattern is compatible with previously described roles of NOTCH signaling in the developing mouse kidney (Cheng et al., 2007; Cheng et al., 2003) and with its role in the development of glomerular disease (Niranjan et al., 2008). Altogether, this analysis identifies signaling pathways that have been described in animal models, as well as new pathways relevant to human kidney development and pathophysiology.

## DISCUSSION

The analysis of single-cell RNA sequencing of human embryonic kidneys provides a powerful tool with which to dissect the gene expression profile during renal organogenesis. The fact that during most of development the kidney contains progenitors and differentiated cells, as well as cells at intermediate developmental stages, precludes the use of conventional high-throughput gene expression techniques. On the other hand, single-cell sequencing is uniquely poised to provide such information. We have used five independent samples and, despite the difference in age between them, they all belong to midpoints in renal development; the histology of the samples confirms the presence of progenitors as well as differentiated cell types (Fig. 1A, Fig. S1H). In addition, all five human developing kidney specimens contribute to each of the clusters (Fig. S1C,D), confirming that those cell types are present in all samples and that our clustering reflects biological differences between these cells rather than sample-to-sample differences.

We have performed an initial clustering followed by sub-clustering of several of the identified populations. This approach has allowed us to investigate the specific gene expression pattern of the developing nephron as shown in Fig. 3D. Although gene expression does not imply a lineage relationship, our data suggest the existence of several intermediate stages in the early developing nephron that are defined by gene expression. Some of these genes present unexpected expression patterns when compared with the mouse developing kidney. For example, both *IRX2* and *IRX3* are co-expressed with *POU3F3* in the distal segment of the s-shaped body; however, in the mouse, they are expressed in the medial segment of the s-shaped body and are absent from the more distal segment (Reggiani et al., 2007). Another example is *HNF4A*, which is also strongly expressed in a subpopulation of *POU3F3* distal cells but, in mice, is restricted to the medial proximal tubule progenitors (Heliot et al., 2013). POU3F3 has been recently identified as a distal marker in both mice and human developing nephrons (Lindstrom et al., 2018b); hence, it is a reliable marker of that segment. Interestingly, the most proximal lineages in the early nephron, i.e. podocytes and parietal epithelial cells, are absent from this sub-clustering. The development trajectory analysis (Fig. 1D) suggests that these are the first cells to express markers that separate them from the nephron progenitor lineage. Hence, the early expression of these genes caused these cells to cluster separately in cluster 4 of our initial analysis and were therefore not included in our sub-clustering analysis of clusters 0-2.

The trajectory analysis has also revealed a significant proximity between lineages that are very distinct in either their origin or their role in development, at least in mouse models. The proximity between the stromal trajectory and the nephron progenitor trajectory reflects a significant degree of gene expression overlap, despite the fact that the epithelial nephron is entirely derived from the nephron progenitors whereas the stroma provides survival cues as well as some non-epithelial cell types. This overlap is likely a reflection of their shared origin: the metanephric mesenchyme. On the other hand, the proximity between the collecting duct and the distal/connecting tubule trajectory reflects the expected proximity in gene expression, as well as the similar functional properties between these two closely located segments, despite originating from distinct renal compartments: the UB and the nephron progenitors, respectively.

The identity of each cluster is assigned based on known gene expression patterns either in human or in mouse developing kidneys; however, new markers for these clusters are also revealed by the analysis. Sub-cluster 1 derived from original clusters 0-2 corresponds to the nephrogenic mesenchyme and is characterized by the expression of *ITGA8*, *EYA1* and *TMEM100*, among other genes (Müller et al., 1997; Rumballe et al., 2011; Xu et al., 1999 and Fig. S1). In addition, we detected strong expression of *COL2A1*, which is a well-established chondrocyte marker (Ng et al., 1993) and *MEG3* (maternally expressed 3) in the nephrogenic mesenchyme. *Col2a1* is expressed in the developing mouse kidney (Wada et al., 1997) but its cell specificity and functional role remain to be elucidated. In humans, mutations in *COL2A1* cause Stickler syndrome, a genetic disorder affecting the connective tissue (Knowlton et al., 1989); however, no renal defects are known to be associated with this syndrome. *MEG3* has not been reported to be expressed in the nephron progenitors and mouse expression in GUDMAP appears to be stromal. Interestingly, *MEG3* is one of several imprinted genes found in sub-cluster 1. A total of five genes specific to the nephrogenic mesenchyme cluster (*H19*, *IGF2*, *MEG3*, *NNAT* and *PTPN14*) are known imprinted genes (Kikyo et al., 1997; Luedi et al., 2007; Miyoshi et al., 2000; Zemel et al., 1992). In addition, *Xist* is imprinted in mice but not in humans (Norris et al., 1994) and *WT1* is imprinted as its alternative form, *AWT1*, in Wilms tumor (Dallosso et al., 2004; Little et al., 1992). Of these, *MEG3*, *NNAT* and *PTPN14* have not been reported to be expressed in the developing kidney and their possible role in nephron progenitors has not been elucidated. Interestingly, Ptpn14 is a negative regulator of Yap (Liu et al., 2013) and Yap is essential for nephron induction and differentiation (Reginensi et al., 2013), and its subcellular localization is dysregulated in Wilms tumor (Murphy et al., 2014).

In the collecting duct (cluster 6 of the initial clustering), the genes most significantly defining the cluster were *GATA3*, *ELF5*, *AGR2*, *ADH1C* and *BCAT1*. GATA3 is a well-known marker of the collecting duct system in mice and human developing kidneys (Labastie et al., 1995; Oosterwegel et al., 1992), and Elf5 has been recently reported in mice as a specific transcription factor in the principal cell lineage (Grassmeyer et al., 2017). On the other hand, the expression of *AGR2*, *ADH1C* and *BCAT1* in the human developing kidney has not been previously reported. CagAgr2 (the fish homolog of AGR2) is expressed in the renal collecting system from gibel carp (Xia et al., 2009) and in the intrahepatic, hilar and extrahepatic biliary tree in embryonic and adult human samples (Lepreux et al., 2011), and Adh1 is expressed in the urothelium of the developing mouse kidney (Mitchell et al., 2006). *BCAT1* (branched chain aminotransferase 1) has not been studied in the kidney nor its expression reported; it has been shown to regulate early liver bud growth in the developing mice and in human embryonic stem cell-derived liver organoids (Koike et al., 2017).

Specific to the immature podocytes (sub-cluster 0 of the sub-clustering of initial cluster 4), we find the expression of olfactomedin 3 (*OLFM3*) (Fig. 3B,C,G). The function of OLFM3 has not been elucidated but it has been suggested to be involved in cell adhesion (Hillier and Vacquier, 2003). Also enriched in the immature podocytes, we find *SLC16A1*, *PCDH9* and *C17orf58* (*1810010H24Rik*). None of these genes has been characterized in the human developing kidney context. Genetic abnormalities in *SLC16A1* have been identified to cause congenital hyperinsulinism (Otonkoski et al., 2007); PCDH9 is a non-clustered protocadherin, mutations in humans are associated with autism spectrum disorders and its expression in glioblastoma suggest a role as tumor suppressor (Kim et al., 2011). On the other hand, mature podocytes (sub-cluster 1 of the sub-clustering of initial cluster 4) show specific expression of netrin G1 (*NTNG1*), a member of the Netrin family. Netrins are extracellular, laminin-related proteins that provide guidance for migrating cells, and NTNG1 is a membrane-tethered glycophosphatidylinositol (GPI)-linked Netrin (Lai Wing Sun et al., 2011). Expression of *NTNG1* has been reported in the adult human kidney by northern blot and semi-quantitative PCR (Meerabux et al., 2005) but its specific podocyte expression in the developing kidney has not been reported. The analysis also unveiled a novel list of PDZ domain proteins expressed in the mature podocytes, suggesting a role for these proteins in the establishment or maintenance of the slit diaphragm in mature human podocytes. This clear distinction between immature and mature podocytes could reflect a transition between pre-functional and functional filtering structures, and the identification of novel markers for each of those populations provides additional tools in the study of podocyte function during development, in homeostasis and during renal disease.

These are just a few examples of the novel cluster-specific genes identified by our single-cell transcriptomics analysis that open new avenues to advance our understanding of human kidney development. Additional studies will be required to characterize the role of these expressed genes, and to determine their functional relevance, possibly through the use of cultured human tissue or via human renal organogenesis.

## MATERIALS AND METHODS

### Single-cell dissociation of human fetal kidney tissue using a cold active protease (subtilisin)

All research using human fetal tissue was approved by the University of Michigan institutional review board. Normal human fetal kidneys at 87, 105, 110, 115 and 132 days of gestation were obtained from the University of Washington Laboratory of Developmental Biology. Gestation age is estimated based on the date of the last period. All tissues were shipped overnight in Belzer's solution at 4°C and were processed immediately upon arrival to the laboratory. Single-cell dissociation was performed using a cold active protease, as described recently (Adam et al., 2017). The embryonic kidneys were decapsulated and cut in half. All tissue samples collected for digestion spanned from the cortical nephrogenic zone to the inner medulla, and were dissected in ice-cold PBS and finely minced in a petri dish on ice using razor blades. About 20 mg of tissue were added to 1 ml of ice-cold active protease solution [PBS, 10 mg of *Bacillus Licheniformis* protease (Sigma, #P5380), 5 mM CaCl_2_, 20 U DNAse I (Roche, #4716728001)]. The tissue was incubated in a 2 ml reaction tube for 15-20 min on a slow-moving shaker (nutator) in a coldroom at 4°C with repeated trituration steps for 20 s every 5 min. Single-cell dissociation was confirmed with a microscope. The dissociation was stopped with 1 ml ice-cold PBS supplemented with 10% fetal bovine serum (FBS). Afterwards, the cells were immediately pelleted at 300 ***g*** for 5 min at 4°C. Subsequently, the supernatant was discarded and cells were suspended in 2 ml PBS/10% FBS and pelleted again at 300 ***g*** for 5 min at 4°C. Then cells were suspended in PBS/0.01% BSA and pelleted again (300 ***g*** for 5 min at 4°C), suspended in 1 ml PBS/0.01%BSA and passed through a 30 µM filter mesh (Miltenyi MACS smart strainer). Viability was then investigated with the Trypan-blue exclusion test and cell concentration was determined using a hemocytometer and adjusted to 200,000 cells/ml for the Drop-seq procedure.

### Drop-seq

Uniformly dispersed 1 nl-sized droplets were generated using self-built polydimethylsiloxane (PDMS) microfluidic co-flow devices on the basis of the AutoCAD design provided by the McCarroll group. The devices were treated with a water repellant solution (Aquapel) to create a hydrophobic channel surface. Drop-Seq runs followed closely the procedure published by Macosko et al. (online Dropseq protocol v. 3.1, mccarrolllab.com/dropseq/). Barcoded beads (ChemGenes), suspended in lysis buffer, were co-flown with a single-cell suspension and a droplet generation mineral oil (QX200, Bio-Rad Laboratories). Resulting droplets were collected in a 50 ml tube and immediately disrupted after adding 30 ml high-salt saline-sodium citrate buffer (6×SSC) and 1 ml perfluorooctanol. Subsequently, captured mRNAs were reverse transcribed for 2 h using 2000 U of the Maxima H Minus Reverse Transcriptase (ThermoFisher) followed by an exonuclease treatment for 45 min to remove unextended primers. After two washing steps with 6× SSC buffer, about 70,000 remaining beads (60% of input beads) were aliquoted (5000 beads per 50 µl reaction) and PCR amplified (five cycles at 65°C and 12 cycles at 67°C annealing temperature). Aliquots of each PCR reaction were pooled and double-purified using 0.5× volume of Agencourt AMPure XP beads (# A63881, Beckman Coulter) and finally eluted in 10 µl EB buffer. Quality and quantity of the amplified cDNAs were analyzed on a BioAnalyzer High Sensitivity DNA Chip (Agilent Technologies). About 600 pg cDNA was fragmented and amplified (17 cycles) to generate a next-generation sequencing library by using the Nextera XT DNA sample preparation kit (Illumina). The libraries were purified, quantified (Agilent High sensitivity DNA chip) and then sequenced (paired end 26×115 bases) on the Illumina HiSeq2500 platform. A custom primer (5′-GCCTGTCCGCGGAAGCAGTGGTATCAACGCAGAGTAC-3′) was used for the first sequence read to identify all different cell barcodes und unique molecular identifier (UMI) sequences.

### Data analysis of scRNASeq data

We processed the sequencing data using the Drop-seq pipeline (mccarrolllab.com/wp-content/uploads/2016/03/Drop-seqAlignmentCookbookv1.2Jan2016.pdf) and generated an expression data matrix. Each element of the matrix is the number of Unique Molecular Identifier (UMIs) associated with a gene in a barcoded cell. Further analysis of the expression matrix was performed using R package Seurat (Butler et al., 2018). We filtered out cells with fewer than 500 genes per cell and with more than 25% mitochondrial read content. The downstream analysis steps include log normalization, identification of highly variable genes across the single cells, scaling, PCA dimensionality reduction, unsupervised clustering and the discovery of differentially expressed cell-type-specific markers. In the scaling step, we regressed out technical variables, including mitochondrial read content, the number of UMI per cell and batch effect using Seurat ScaleData function. We used the default non-parametric Wilcoxon rank sum test for the differentially expression analysis. The clusters were viewed in a two-dimensional frame using tSNE clustering (Shekhar et al., 2016). The initial unsupervised clustering was performed at a resolution level of 0.6 and it resulted in 11 clusters. Next, we did sub-clustering on individual cell clusters. In the sub-clustering analysis, we regressed out one more parameter, cell cycle gene expression effect. The list of the cell cycle genes was downloaded from satijalab.org/seurat/cell_cycle_vignette.html. We used 0.6 as resolution for all of the unsupervised sub-clustering analyses. As the markers of cell clusters 0, 1 and 2 indicated these cell types to be less differentiated than the other cell clusters, we carried out sub-clustering of these three clusters together. The R package Monocle (Trapnell and Cacchiarelli, 2014) was used to find the expression dynamics of the signaling molecules across sub-clusters of the initial clusters. Cells with more than 1000 genes and the set of variable genes (genes that are more than one standard deviation apart from the average dispersion within a bin) from the Seurat sub-clustering analyses were used to order the cells using Monocle. Gene Ontology enrichment analysis was performed on the cluster-specific differentially expressed genes from the initial clustering analysis using iPathwayGuide (apps.advaitabio.com/oauth-provider/).

#### Ligand-receptor interaction

We used receptors and ligand pairs from the public resource Database of Interacting Partners (dip.doe-mbi.ucla.edu/dip/DLRP.cgi). Any ligand or receptor was classified as expressed if it was significantly differentially in a cell cluster (adjusted *P* value<0.001). Owing to extremely high or low cell numbers, to be more accurate, the expression of the corresponding partner had to be greater than the mean expression across all clusters.

#### Correlation analysis

The postnatal day 1 mouse kidney scRNASeq dataset (GSE94333) (Adam et al., 2017) was downloaded from NCBI GEO (www.ncbi.nlm.nih.gov/geo/query/acc.cgi?acc=GSE94333) and analyzed in the same way as the human fetal kidney using the Seurat R package. All clusters published by the authors have been identified. In addition, we found an immune cell cluster with 76 distinct transcriptomes. Correlation analysis was performed on the average gene expression in all 11 clusters from the initial unsupervised clustering of the human fetal kidney data with the clusters of postnatal day 1 mouse kidney data.

### Trajectory estimation from single-cell RNA-seq data

Single-cell RNA-seq data was normalized and log-transformed by R package Seurat. The transformed data was filtered by the following criteria: (1) include only genes with detected expression in at least three cells; and (2) include only cells with at least 1000 genes detected and with less than 5% of total reads being mitochondria reads.

To remove technical variations, we regressed out the number of genes expressed, the percentage of mitochondria reads and batch variables via a linear model. To control for confounding effects of cell cycle to trajectory estimation, we normalized expression levels of non-cell cycle genes to a baseline estimated using expression of only cell cycle genes. Specifically, we used a curated cell cycle gene list of 1946 genes from Barron and Li (2016); for each non-cell cycle gene, we fitted a ridge regression model to predict its log-transformed expression level from cell-cycle genes, using the cv.glmnet function in R package glmnet. The regularization parameter was automatically selected based on 10-fold crossvalidation. With the fitted models, we computed the residuals of each non-cell cycle gene and used the residuals as input for trajectory analysis.

Trajectory analysis was performed with a method described by J.Z. and O.T. (unpublished) that uses a nonparametric estimation method based on probability distribution. To identify genes with significant expression change along the trajectory, for each trajectory segment of interest, we tested the significance of each gene by fitting a generalized additive model (GAM) with thin plate regression spline to predict expression level from the cell order in the trajectory segment using the gam function of R package mgcv. The significance level of the gene is the significance level of cell order in the GAM model.

### Immunofluorescence

Kidneys were fixed overnight in 4% PFA and processed for frozen sectioning and incubation as previously reported (Cebrián, 2012). Sections (5 μm) were incubated at 4°C overnight with primary antibodies against the following epitopes: decorin (R&D systems, AF143), ETV4 (Proteintech, 10684-1-AP), PAX8 (Proteintech, 10336-1-AP), RET (R&D, AF1485), synaptopodin (Proteintech, 21064-1-AP) and tenascin C (R&D, MAB2138). Alexa 488-conjugated secondary antibodies were purchased from Jackson ImmunoResearch (705-546-147, 711-546-152 and 712-546-153) and incubated at 37°C for 3 h. All primary and secondary antibodies were used at 1:100 dilution. Imaging was performed on an Olympus IX71 inverted microscope or a Nikon A1 confocal microscope.

## Supplementary Material

Supplementary information
