# Supplementary material for: Single-cell analysis of progenitor cell dynamics and lineage specification in the human fetal kidney
Source: Development. 2018 Aug 30;145(16):dev164038. doi: 10.1242/dev.164038 (PMC6124540; doi:10.1242/dev.164038)
Supplement: Supplementary information [file develop-145-164038-s1.pdf]

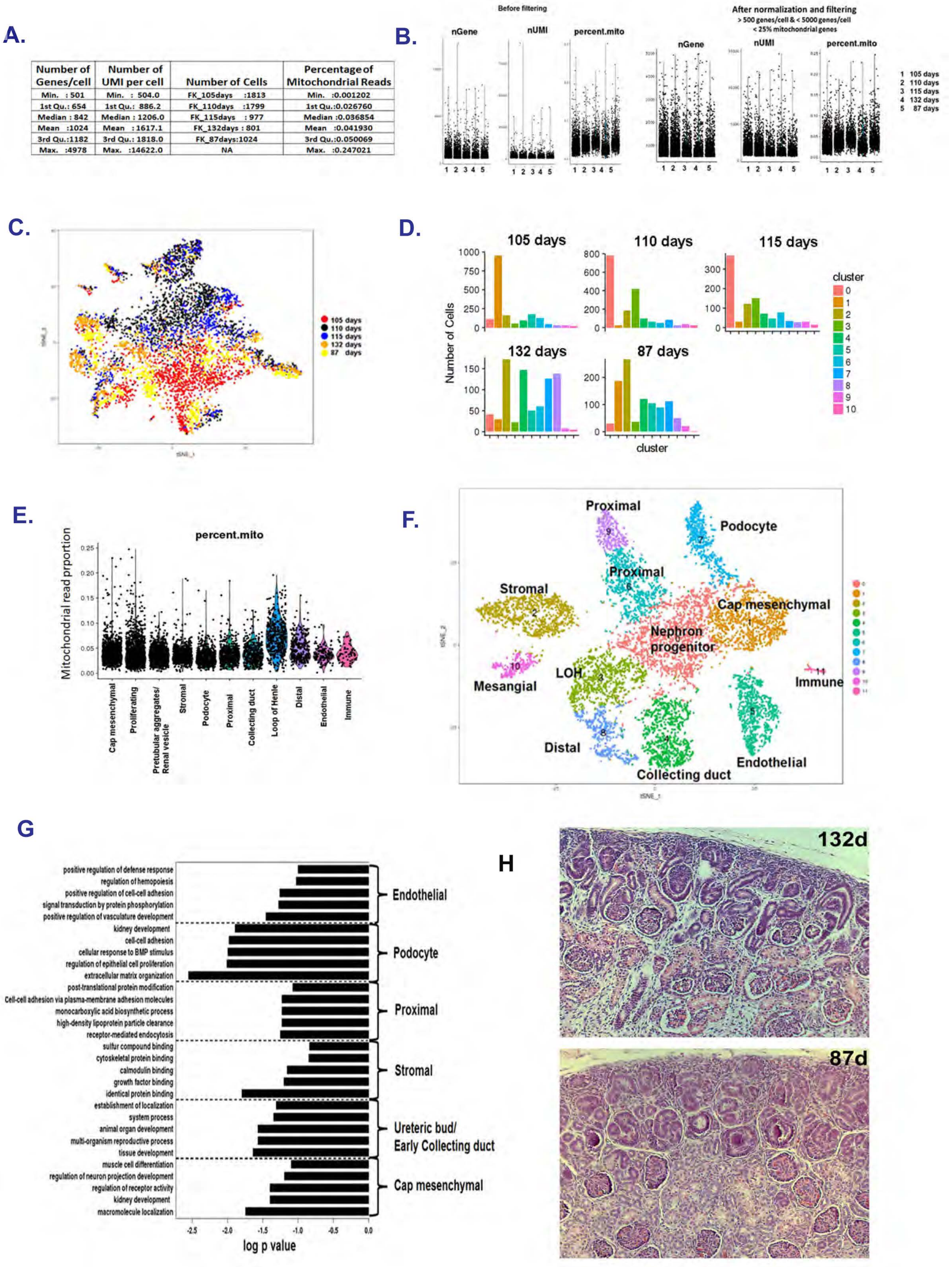

Number of Genes/cell	Number of UMI per cell	Number of Cells	Percentage of Mitochondrial Reads
Min. : 501	Min. : 504.0	FK_105days : 1813	Min. : 0.001202
1st Qu.: 654	1st Qu.: 886.2	FK_110days : 1799	1st Qu.: 0.026760
Median : 842	Median : 1206.0	FK_115days : 977	Median : 0.036854
Mean : 1024	Mean : 1617.1	FK_132days : 801	Mean : 0.041930
3rd Qu.: 1182	3rd Qu.: 1818.0	FK_87days: 1024	3rd Qu.: 0.050069
Max. : 4978	Max. : 14622.0	NA	Max. : 0.247021

Development • Supplementary information

Figure S1: A) Table showing the summary of the quality control (QC) parameters including number of genes/cell (nGene), number of unique molecular identifier (nUMI) and mitochondrial read content for the 5 fetal kidney scRNASeq datasets **B)** Violin plots of the QC parameters in the 5 datasets before and after filtering out cells with < 500 genes and > 25% mitochondrial read content. **C)** tSNE plot depicting the distribution of cells from the 5 fetal kidney datasets in the 11 clusters from the initial unsupervised clustering. **D)** Bar plots depicting the contribution of the cells from the 5 fetal kidney datasets in the 11 clusters from the initial clustering **E)** Violin plots of mitochondrial read content per cell in the 11 clusters from the initial clustering. . **F)** tSNE plot showing the clusters from the unsupervised clustering of the postnatal day 1 mouse kidney samples (GSE94333). **G)** Top 5 Gene Ontology biological processes for the cluster-specific significantly differentially expressed ($p < 0.05$) genes of 11 clusters from the initial clustering. **H)** H&E staining of thin paraffin sections of human embryonic kidneys of 87 and 132 days post period.

Development • Supplementary information

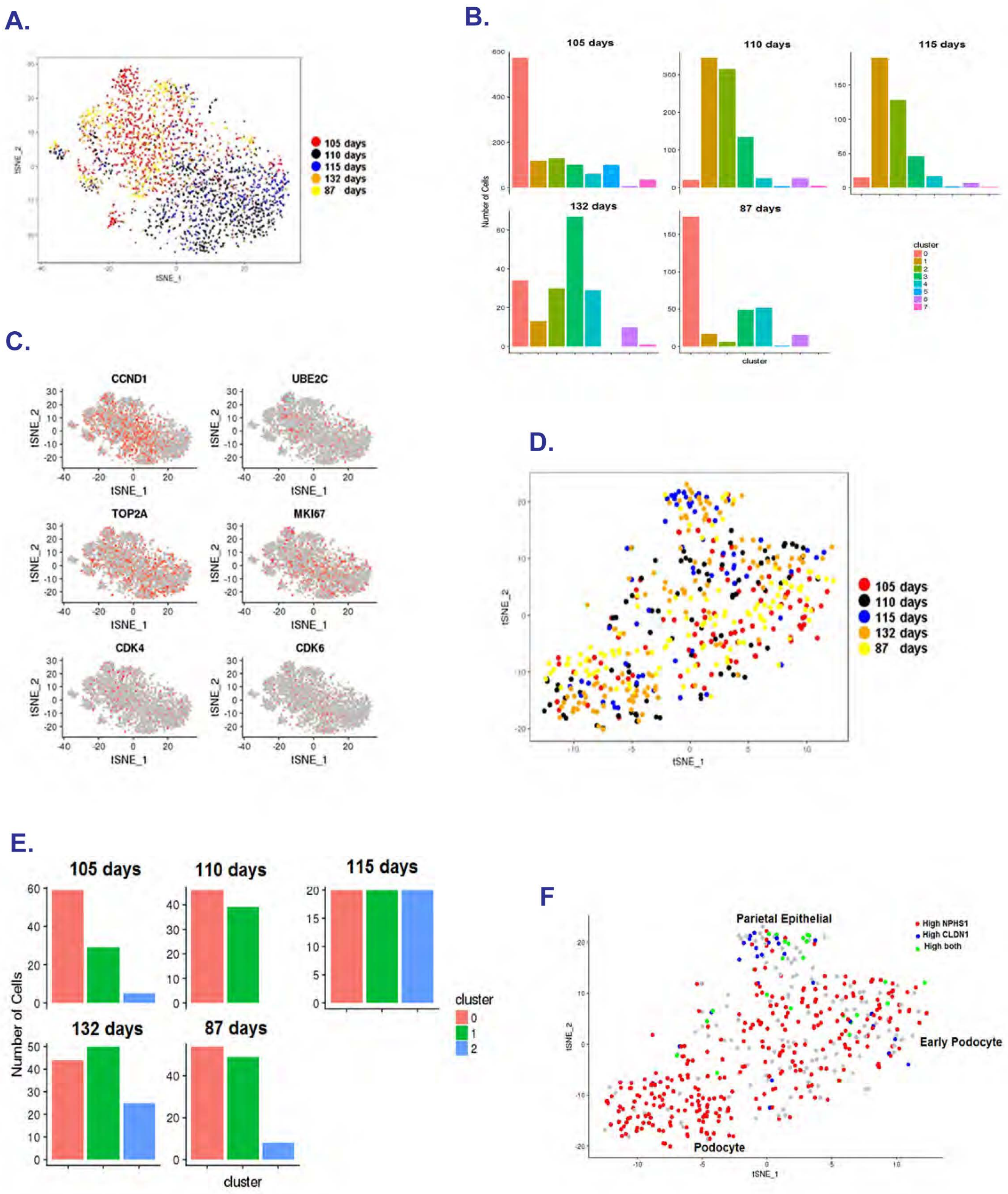

Development • Supplementary information

Figure S2: A) tSNE plot depicting the distribution of cells from the 5 fetal kidney datasets in the 8 sub-clusters of initial clusters 0, 1 and 2 **B)** Bar plots depicting the contribution of the cells from the 5 fetal kidney datasets in the 8 sub-clusters from the initial clusters 0,1 and 2. **C)** Feature plot of proliferation-related genes including CCND1, UBE2C, TOP2A, MKI67, CDK4 and CDK6 in the sub-clustering from the initial unsupervised clustering of clusters 0,1 and 2. **D)** tSNE plot depicting the distribution of cells from the 5 fetal kidney datasets in the 3 sub-clusters of cluster 4 from the initial unsupervised clustering. **E)** Bar plots depicting the contribution of the cells from the 5 fetal kidney datasets in the 3 sub-clusters from the initial cluster 4. **F)** Feature plot of NPHS1, CLDN1 expression in the three sub-clusters of original cluster 4. Cells with high expression of NPHS1 are in red and the cells with high expression of CLDN1 are in blue. Cells with high expression of both NPHS1 and CLDN1 are in green.

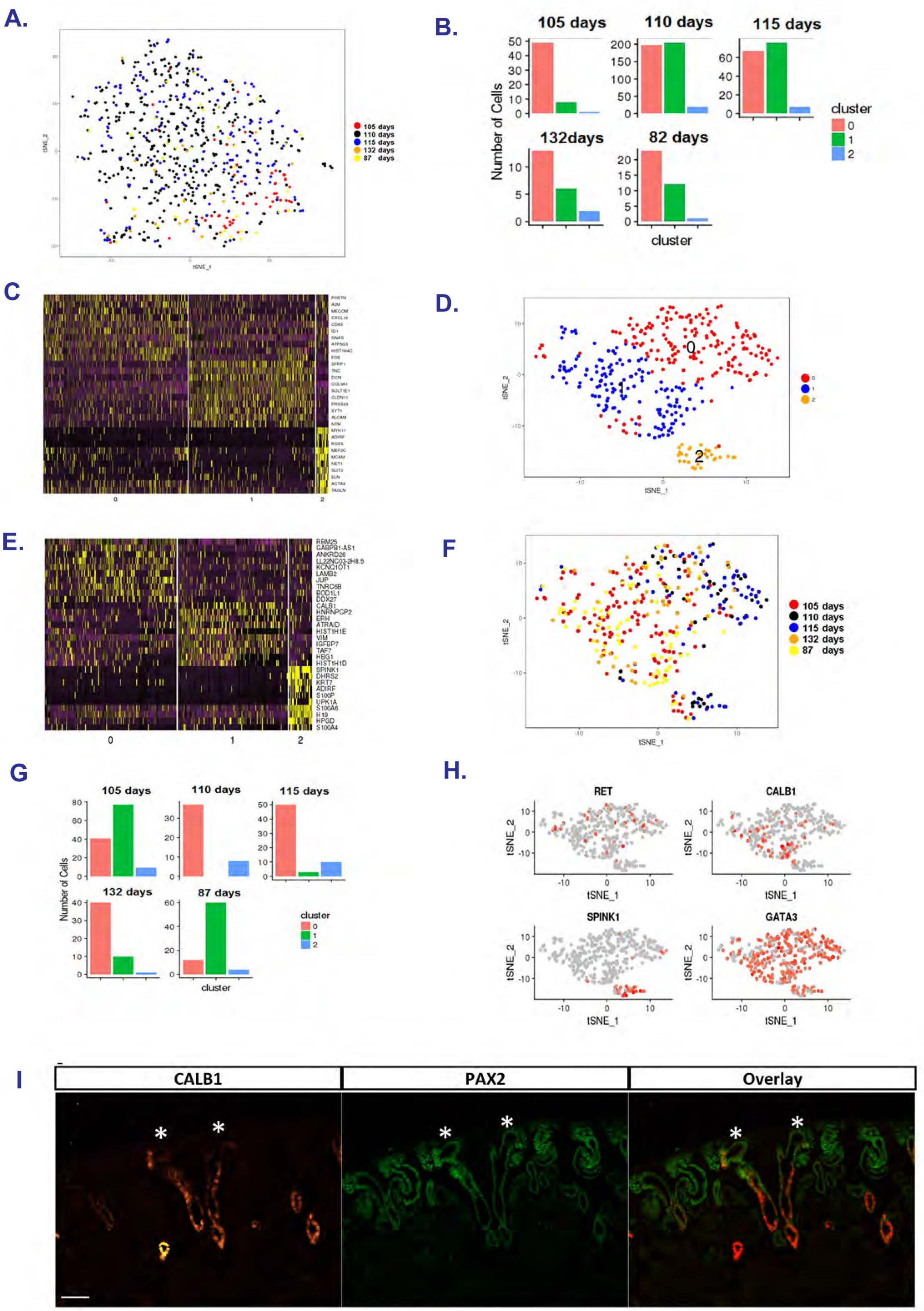

Development • Supplementary information

Figure S3: A) tSNE plot depicting the distribution of cells from the 5 fetal kidney datasets in the 3 sub-clusters of cluster 3 (Stromal) from the initial unsupervised clustering. **B)** Bar plots depicting the contribution of the cells from the 5 fetal kidney datasets in the 3 sub-clusters from the initial cluster 3 (Stromal) **C)** Heatmap with the expression of the top 10 cluster-specific genes in the 3 sub-clusters of the original cluster 3 (Stromal). **D)** tSNE plot showing the three sub-clusters of cluster 6 (UB) from the initial clustering. **E)** Heatmap with the expression of the top 10 cluster-specific genes in the 3 sub-clusters of the original cluster 6 (UB). **F)** tSNE plot depicting the distribution of cells from the 5 fetal kidney datasets in the 3 sub-clusters of cluster 6 (UB) from the initial unsupervised clustering. **G)** Bar plots depicting the contribution of the cells from the 5 fetal kidney datasets in the 3 sub-clusters from the initial cluster 6 (UB) **H)** Expression of known markers (RET, CALB1, SPINK1, GATA3) in cluster 6 (UB) sub-clusters. **(I)** Immunofluorescent staining of human embryonic sections with anti-Calbindin1 and anti-PAX2 antibodies. Calbindin staining is excluded from the tips of the ureteric buds (asterisks). Scale bar: 100um. Representative image of 3 independent stainings.

A

Significantly differentially expressed gene (ligand/receptor)	cluster	Corresponding interactor (ligand/receptor)	cluster (> mean expression across all clusters)
BMP7	4	ACVR1	0, 3
EFNB2	4, 6, 9	EPHB3	0, 2, 7, 8
EPHA7	5	EPHB3	0, 2, 7, 8
ERBB2	8	NRG1	4, 6, 7
ERBB4	2, 7, 8	BTC	4, 5, 6
FLT1	9	VEGFC	4, 8, 9
IGF2	0, 3	IGF2R	4, 7, 8, 9
IL6ST	9	CTF1	2, 4, 7, 8
JAG1	5	NOTCH4	9
JAG1	5	NOTCH2	0, 3, 4
KDR	9	VEGFC	4, 8, 9
LIFR	9	CTF1	2, 4, 7, 8
MDK	0, 3	PTPRB	9
NOTCH4	9	JAG1	0, 1, 2, 5, 8
NTRK2	0	BDNF	0, 3, 4, 5, 6
PTN	3	PTPRB	9
TGFBR2	9	TGFB3	0, 3, 6
TGFBR2	9	TGFB2	0, 3
TGFBR3	4	TGFB3	0, 3, 6, 9
TGFBR3	4	TGFB2	0, 3
TNFSF10	5	TNFRSF10B	2, 4, 5, 7, 8

B

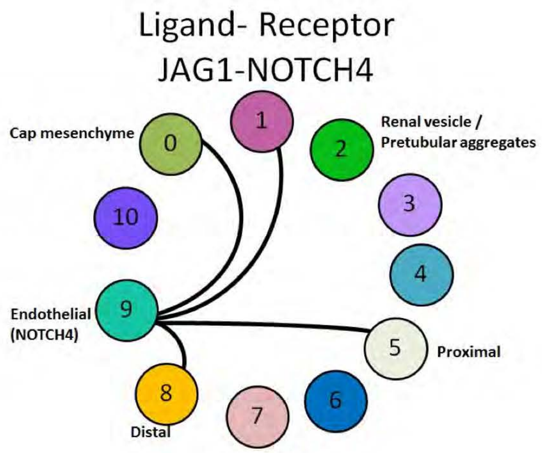

Figure S4: A) Table showing the list of either ligand or receptor that was significantly differentially expressed in a cell cluster compared to all other clusters (adjusted p value < 0.001). The corresponding interacting partner expression is greater than the mean expression across all other clusters. **B)** The figure illustrates the possible interaction of ligand JAG1-NOTCH4 interaction. NOTCH4 expression was specifically seen in endothelial cell cluster.

Development • Supplementary information

Supplementary Tables

Table S1: The table provides list of significantly expressed genes for each of the 11 clusters compared to all other clusters. pct.1 is the proportion of cells expressing the gene in the cluster of interest and pct.2 is the proportion of cells expressing the gene in all other clusters. avg_logFC is the log fold-change of the average expression between the cluster of interest and all other clusters. Positive values indicate that the gene is more highly expressed in the first group.

Click here to Download Table S1

Table S2: The cells from the progenitor cell clusters (clusters 0, 1 and 2) in the first clustering were further sub-clustered. The table provides list of significantly expressed genes for each of the 8 sub-clusters compared to all other sub-clusters. pct.1 is the proportion of cells expressing the gene in the cluster of interest and pct.2 is the proportion of cells expressing the gene in all other clusters. avg_logFC is the log fold-change of the average expression between the cluster of interest and all other clusters. Positive values indicate that the gene is more highly expressed in the first group.

Click here to Download Table S2

Table S3: The cells from the podocyte-like cluster (cluster 4) in the first clustering were further sub-clustered. The table provides list of significantly expressed genes for each of the 3 sub-clusters compared to all other sub-clusters. pct.1 is the proportion of cells expressing the gene in the cluster of interest and pct.2 is the proportion of cells expressing the gene in all other clusters. avg_logFC is the log fold-change of the average expression between the cluster of interest and all other clusters. Positive values indicate that the gene is more highly expressed in the first group.

Click here to Download Table S3

Table S4: The cells from the stromal cluster (cluster 3) in the first clustering were further sub-clustered. The table provides list of significantly expressed genes for each of the 3 sub-clusters compared to all other sub-clusters. pct.1 is the proportion of cells expressing the gene in the cluster of interest and pct.2 is the proportion of cells expressing the gene in all other clusters. avg_logFC is the log fold-change of the average expression between the cluster of interest and all other clusters. Positive values indicate that the gene is more highly expressed in the first group.

Click here to Download Table S4

Table S5: The cells from the collecting duct-like cluster (cluster 6) in the first clustering were further sub-clustered. The table provides list of significantly expressed genes for each of the 3 sub-clusters compared to all other sub-clusters. pct.1 is the proportion of cells expressing the gene in the cluster of interest and pct.2 is the proportion of cells expressing the gene in all other clusters. avg_logFC is the log fold-change of the average expression between the cluster of interest and all other clusters. Positive values indicate that the gene is more highly expressed in the first group.

Click here to Download Table S5

Development • Supplementary information

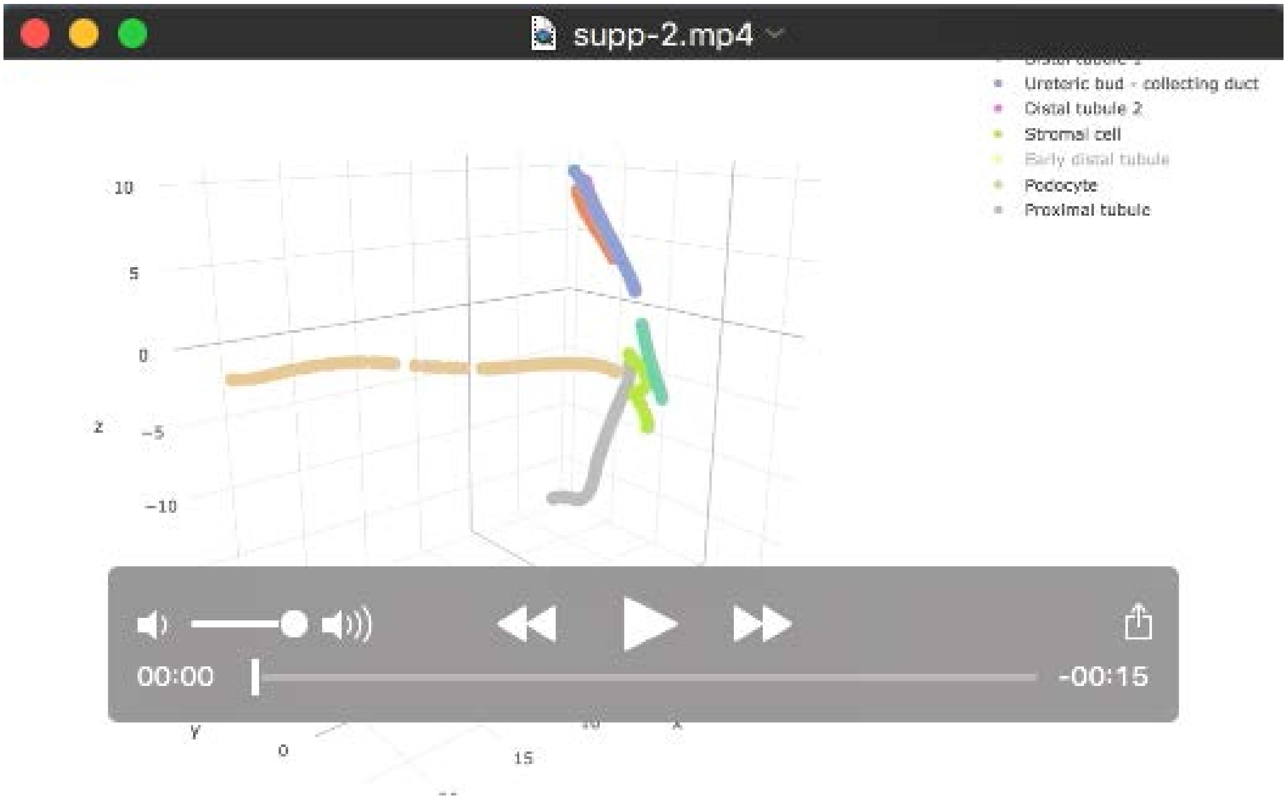

Movie 1: 3-Dimensional view of the pseudo-temporal trajectory analysis of the fetal kidney single cell expression data. Single cell RNASeq data from 5 human fetal kidney datasets (87, 105, 110, 115, and 132 days old) were used in this analysis. Each developmental trajectory is color-coded and each dot represents a cell. Cells with > 1000 genes /cell and < 25% mitochondrial read content were used this cell-ordering analysis. 3 independent developmental trajectories were identified: the stroma, the collecting duct and the nephron lineage. The proximity between the stromal trajectory and the nephron progenitor trajectory reflects a significant degree of gene expression overlap. Similarly, the proximity between the collecting duct and the distal/connecting tubules trajectory reflects the expected proximity in gene expression, as well as function between these two closely located segments, despite originating from distinct renal compartments, the UB and the nephron progenitors respectively.
